# Automated Surveillance of Ventilator-Associated Events and Mortality Before and During the COVID-19 Pandemic: A Single-Center Retrospective Study in Japan

**DOI:** 10.7759/cureus.110846

**Published:** 2026-06-14

**Authors:** Junichi Yoshida, Kenichiro Shiraishi, Akiko Mataga, Tetsuya Kikuchi, Masao Tanaka

**Affiliations:** 1 Office of Infection Control, Shimonoseki City Hospital, Shimonoseki, JPN

**Keywords:** covid-19, mortality, pneumonia, season, ventilator-associated event

## Abstract

Purpose

Using in-house automated surveillance of ventilator-associated events (VAE) from our electronic health record (EHR) system, we investigated clinical and microbiological factors associated with mortality among patients receiving mechanical ventilation (MV). Our primary objective was to identify risk factors for mortality in patients during MV. Our secondary objectives included assessing the impact of COVID-19 and examining any seasonal variations in mortality during MV.

Methods

All patients who received MV between 2013 and 2025 were included. The internal review board approved the study as a retrospective analysis in 2026. We defined cold seasons as October through March. Using a straightforward VAE calculator in accordance with the Centers for Disease Control and Prevention’s guidelines, the primary outcome was all-cause mortality during MV. We excluded deaths after extubation to study patients undergoing MV alone. Patients with missing data due to historical changes in documentation were analyzed collectively in a subgroup analysis.

Results

A total of 17,881 ventilator days were recorded among 2,003 patients. Multivariate analysis indicated that significant risk factors for mortality included age ≥ 75 (odds ratio [OR] 1.343, 95% confidence interval [95% CI] 1.006-1.792, P = 0.045) and the presence of ventilator-associated conditions (OR 1.926, 95% CI 1.017-3.645, P = 0.044). Cold seasons were associated with lower mortality during MV (OR 0.752, 95% CI 0.572-0.987, P = 0.040). Additionally, there was a marginal association between cold seasons and carbapenem use (P = 0.07), a class of broad-spectrum antimicrobials. Notably, April exhibited the highest mortality during MV, coinciding with a change in physician staffing, with a median turnover of 27.1% among all the house staff.

Neither the pandemic era (OR 1.059, 95% CI 0.801-1.400, P = 0.689) nor SARS-CoV-2 positivity (OR 1.211, 95% CI 0.525-2.793, P = 0.654) was found to be associated with mortality.

Conclusions

As to the primary objectives, we identified ventilator-associated condition, the first of three tiers of VAE, as a risk factor for mortality during MV.

Regarding the secondary objectives, we found that the COVID-19 pandemic and SARS-CoV-2 positivity were not associated with mortality during MV. Meanwhile, mortality during MV was associated with seasonal factors; however, whether antimicrobial prescribing patterns or physician turnover contributed requires further study.

## Introduction

The current definition of ventilator-associated pneumonia surveillance, as established by the Centers for Disease Control and Prevention (CDC) in the USA, classifies ventilator-associated events (VAE) into three tiers [1]. These tiers were developed to minimize clinical diagnoses of ventilator-associated pneumonia as follows: 1) ventilator-associated condition (VAC) is defined based on the settings of mechanical ventilation (MV); 2) infection-related ventilator-associated complication (IVAC) is defined as the presence of infection that requires treatment; and 3) probable ventilator-associated pneumonia (PVAP) is characterized by laboratory evidence.

We conducted a retrospective study to identify risk factors for mortality during MV. To facilitate this analysis, we developed an automated in-house VAE system based on the CDC's internet VAE Calculator [2]. This calculator allows for immediate computation once ventilator parameters are input. However, transferring data requires manual entry online, which can be time-consuming and prone to errors. By automating this calculation within our in-house system, we aim to eliminate the need for manual entry and improve accuracy.

One of the major infectious disease challenges in recent years has been the COVID-19 pandemic caused by SARS-CoV-2, which has since become endemic in Japan [3]. The pandemic's impact on healthcare-associated infections warrants further evaluation. Specifically, it is important to assess mortality during MV through surveillance. Our teaching hospital has access to a large volume of electronic health records (EHR), which is beneficial for retrospective EHR analysis.

Regarding seasonality in Japan, physician transitions occur in April, unlike other countries, where transitions typically take place in July. This could lead to changes in treatment for patients undergoing MV. We hypothesized that there may be a seasonal variation in mortality during MV.

In this study, our primary objective was to investigate risk factors for mortality during MV. Our secondary objectives included assessing the influence of COVID-19 and hypothetical seasonal effects on mortality during MV.

## Materials and methods

Patients

All patients who received MV between 2013 and 2025 were included. Eligibility criteria included MV in the intensive care and other wards of our teaching hospital. Inclusion criteria were invasive MV via endotracheal intubation and tracheostomy. Exclusion criteria included high-flow nasal oxygen therapy and non-invasive ventilation.

Methods

This study was a retrospective, single-center observational study. Data relevant to VAE were extracted from EHR using HOPE LifeMark-HX (Fujitsu Limited, Kawasaki, Japan). The data included parameters such as positive end-expiratory pressure (PEEP), fraction of inspired oxygen (FiO_2_), body temperature, administered antimicrobials, airway-derived bacteria, and viral tests (e.g., influenza and SARS-CoV-2), and white blood cell counts during MV. These data were collected as daily values, worst values per day, and intermittent measurements within a single day.

Demographic data were obtained on age, sex, and admission diagnoses. The COVID-19 pandemic era was defined as the years 2020 through 2025. This era was further divided into three distinct periods: the early pandemic era in 2020, the vaccination era in 2021, and the Omicron/endemic era from 2022 to 2025.

Cold seasons were defined as MV beginning between October and March. In Japan, academic seasons begin in April, coinciding with the transition of residents and specialty physicians to new hospitals. We documented the monthly tracking of physician changes.

Additional data included the sepsis-related organ failure assessment (SOFA) score, defined as the sum of differences from baseline scores [4]. The EHR, however, documented SOFA scores in recent years. The Charlson Comorbidity Index (CCI) [5] was calculated for patients undergoing MV. We adopted CCI for the assessment of diagnosis severity and reasons for ICU admission because of the lack of standardized methods for quantifying severity across various diseases. The duration of MV days was recorded, along with the presence or absence of emergency operations. Any treatment limitations were also documented.

Patients with missing values were included in the collective subgroup analysis, ensuring that those with historical changes in documentation were taken into account as well.

In preparing this work, the authors first used generative artificial intelligence (AI) from Gemini (Alphabet Inc., Mountain View, CA, USA) to assist in coding the CDC protocol. We implemented the data from the EHR within our in-house system and evaluated its effectiveness.

Alternatively, we developed an in-house VAE calculator using Visual Basic for Applications (VBA; Microsoft Corp., Redmond, WA, USA) within Excel (Microsoft). Its algorithm adhered to the adult protocol described in Chapter Definitions (pages 3-14) of the CDC document [1]. Serial measurements of PEEP, FiO_2_, white blood cell counts, and body temperature were calculated on a given day to assess VAE. When patients met the criteria for VAC, antimicrobial data were incorporated for IVAC, and subsequently, microbiological results were added for PVAP.

These data comprised daily values, worst values per day, and intermittent measurements within a day. All the data were extracted from the EHR into Excel using the in-house system. Macro programs coded in VBA were then applied to produce outcomes such as VAC, IVAC, and PVAP. Some of the generated outcomes were validated against the CDC Calculator to ensure their accuracy and acceptance.

Within the in-house system, all the data were extracted from the EHR into Excel, underwent macro programs coded using VBA, and produced outcomes of VAC, IVAC, and PVAP. Part of the generated outcomes were validated against the CDC Calculator to ensure their acceptance or rejection. 

Statistics

For the statistical procedures, we conducted univariate logistic regression analysis to assess mortality during MV. All variables that had no missing values proceeded to multivariate logistic regression analysis. Consequently, a primary group of complete variables underwent this multivariate analysis. In a subgroup analysis, we included additional complete variables in the multivariate analysis. Background factors encompassed a range of elements specified in the VAE protocol [1] and various demographic indices. According to the bacterial culture protocol, we included bacterial species with colony-forming units ≥ 5 per microliter, while excluding colonization or fungal species [1]. 

We examined the antimicrobial factors of the last drugs prescribed during MV to investigate the microbiological context prior to mortality during MV. Initial antimicrobial treatments were mostly empiric, and there were frequent de-escalations or changes to treat drug-resistant microbes. Additionally, we analyzed time spans, focusing on either the last 24 hours, the last 48 hours, or the time of extubation or mortality during MV. For the forest plots illustrating odds ratios related to mortality during MV, we utilized Excel software.

For any factors associated with increased mortality during MV, we calculated correlation coefficients to explore mutual relationships. In our statistical evaluations, we considered P < 0.05 to be significant. We employed SPSS Version 31 (IBM Corporation, Armonk, NY, USA) for all statistical calculations.

Ethics

The authors obtained ethical approval from the Internal Review Board of the institute with permission number 2026SCHEC-36-2 to conduct the study. The requirement for informed consent was waived as per "Chapter 5, Part 12, B Research not involving invasiveness" of the Ethical Guidelines issued by the Ministry of Education, Culture, Sports, Science and Technology, Japan [6].

## Results

Outline

**Figure 1:**
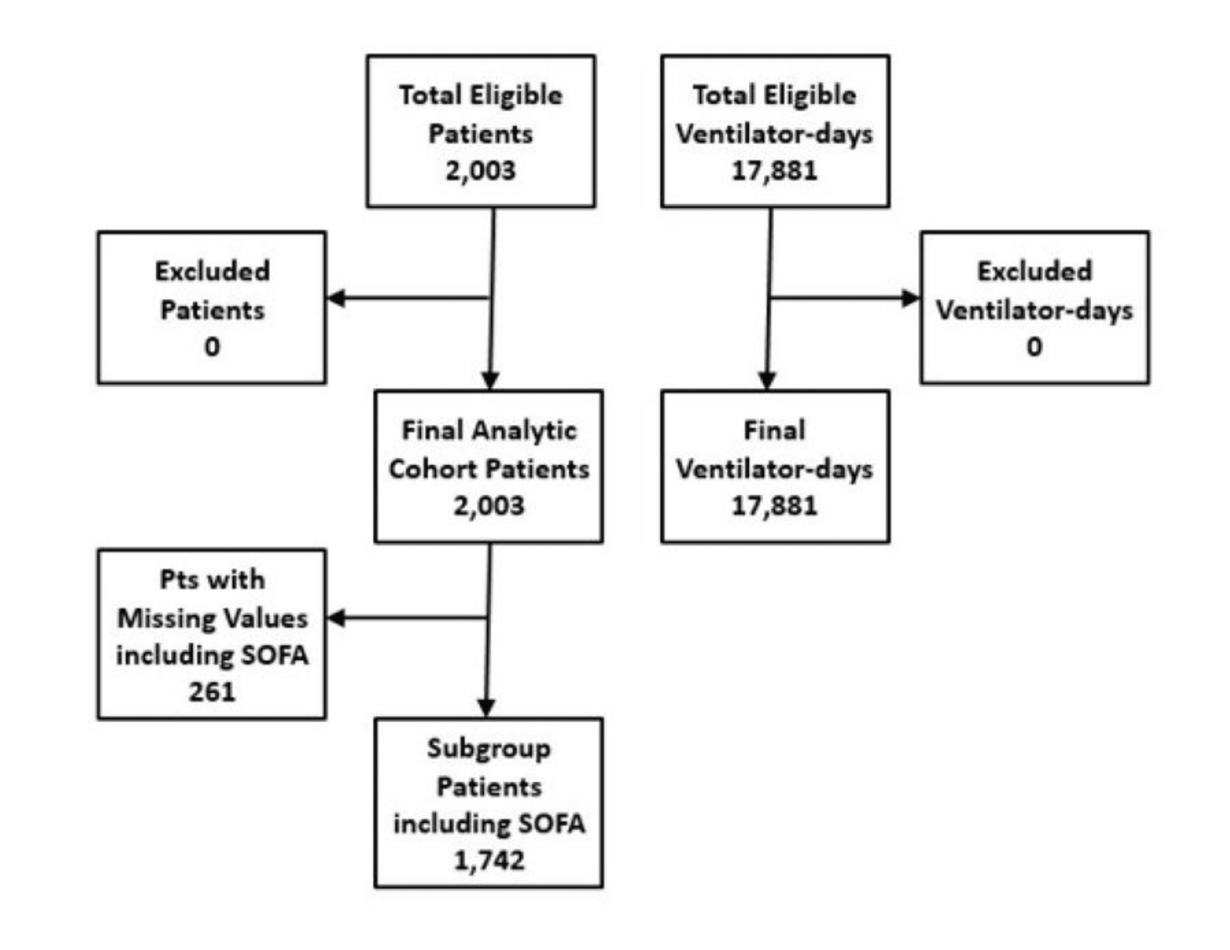
Flow diagram of patients and ventilation days in the current study. The final analytic cohort of patients and ventilation days is analyzed. The subgroup with missing values, including sepsis-related organ failure assessment (SOFA) scores, underwent further analysis.

Among all patients, values for PEEP, FiO_2_, temperature, white blood cell counts, microbiology, and antimicrobial treatments were complete, except for the additional data outlined in the Methods section. Consequently, a total of 17,881 MV days across 2,003 patients were included in the final cohort analysis. Additionally, a subgroup analysis was performed on 1,742 patients who had missing values in the additional data (Figure 1).

As a limitation of the treatment, the final group included only two patients diagnosed with COVID-19, who were subsequently transferred to university hospitals for specialized treatment. The admission diagnosis of pneumonia accounted for the highest number of cases, with 253 patients (12.6%) (Table 1).

**Table 1:** Diagnoses of patients (N=2,003) undergoing mechanical ventilation. Note: %, percentage (allowing for rounding errors).

Diagnosis	N	%	Category	N	%
Abdominal Diseases	6	0.3	Abdomen	250	12.5
Bile Duct Diseases	38	1.9			
Intestinal Diseases	143	7.1			
Pancreatic Diseases	6	0.3			
Peritoneal Diseases	57	2.8			
Malignancy	220	11.0	Cancer	220	11.0
Cardiovascular Diseases	680	33.9	Circulation	680	33.9
Orthopedic Diseases	94	4.7	Extremities	115	5.7
Dermatological Diseases	4	0.2			
Soft Tissue Diseases	17	0.8			
Hematological Diseases	16	0.8	Hematology	16	0.8
Ear/Nose/Throat Diseases	16	0.8	Head/Neck	22	1.1
Eye Diseases	3	0.1			
Oral Diseases	3	0.1			
Brain Diseases	233	11.6	Neurology	233	11.6
Asphyxia	5	0.2	Respiration	310	15.5
Chest Diseases	45	2.2			
Pneumonia	253	12.6			
Tracheal Diseases	7	0.3			
Autoimmune Diseases	4	0.2	Systemic	53	2.6
Diabetes Mellitus	6	0.3			
Metabolic Diseases	40	2.0			
Intoxication	3	0.1			
Trauma	28	1.4	Trauma	28	1.4
Renal Diseases	16	0.8	Urology	76	3.8
Septic Diseases	40	2.0			
Urinary Tract Diseases	20	1.0			
Total	2,003	100.0		2,003	100.0

We initially used Gemini for in-house calculations of VAE to create VBA code in Excel. However, the dataset extracted from our EHR was not suitable for VAE calculations due to a computational mismatch. Consequently, we developed our own in-house VAE calculator.

To validate the accuracy of our in-house system, we analyzed a total of 84 VACs (Table 2) using the CDC Calculator, which confirmed VACs in all 84 cases. In contrast, data from a randomly sampled 100 patients identified with negative VAEs were input into the CDC Calculator, and all returned as negative. However, this manual chart abstraction and input into the online calculator required data transfer from the in-house system. Our system administrator indicated that this transfer would take one to two business days, which made manual data searches impractical.

**Table 2:** Results of ventilator-associated events (VAE) in three tiers. For a total of 17,881 mechanical ventilation (MV) days, rates are shown per 1,000 days (permille). Note: VAC, ventilator-associated condition; IVAC, infection-related ventilator-associated complication; PVAP, probable ventilator-associated pneumonia; #, antimicrobials administered last during MV.

Outcome	Survival		Deaths		Total	
Ventilator Days	N=15,110	permille	N=2,771	permille	N=17,881	permille
VAC	65	4.3	19	6.9	84	4.7
IVAC	17	1.1	5	1.8	22	1.2
PVAP	3	0.2	3	1.1	6	0.3

For the treatment of methicillin-resistant *Staphylococcus aureus* (MRSA), we utilized vancomycin, linezolid, and daptomycin. In cases of pneumonia, we administered vancomycin and linezolid. Among the last antimicrobials given during MV positive for VAE (Table 3), MRSA treatments included vancomycin (N=4) and daptomycin (N=1). None of these drugs was given for pneumonia.

**Table 3:** Breakdown of demographic and clinical factors by outcome of survival or deaths during mechanical ventilation. The upper part of the table is for the final group of patients (N=2,003) and the lower part is for the subgroup of patients (N=1,742). Rates are shown as percentages (%), permitting rounding errors. Note: M, male; F, female; MRSA, methicillin-resistant *Staphylococcus aureus*; MRSA drugs, including vancomycin and daptomycin; ESBL, extended-spectrum beta-lactamase production; *P. aeruginosa*, *Pseudomonas aeruginosa*; *S. maltophilia*, *Stenotrophomonas maltophilia*; PCs, penicillins; SOFA, sepsis-related organ failure assessment; $, applied with Charlson Comorbidity Index (CCI) for main diagnoses; MV, mechanical ventilation; EmOp, emergency operation.

Outcome	Survival		Deaths		Total	
Main group		%		%		%
Patients	1,756	87.7	247	12.3	2,003	100.0
	Median	(Quartile)	Median	(Quartile)	Median	(Quartile)
Age	78	(69-84)	77	(69-84)	78	(69-84)
Sex: Male with Female as Reference	M: 1,058	F: 698	M: 151	F: 96	M: 1,209	F: 794
Intensive Care	1,446	72.2	183	9.1	1,629	81.3
Tracheostomy	226	11.3	25	1.2	251	12.5
Prone Position	28	1.4	9	0.4	37	1.8
Pandemic	915	45.7	135	6.7	1,050	52.4
COVID-19	36	1.8	8	0.4	44	2.2
Influenza	14	0.7	6	0.3	20	1.0
Cold Season	919	45.9	114	5.7	1,033	51.6
MRSA	37	1.8	4	0.2	41	2.0
ESBL	5	0.2	1	0.0	6	0.3
P. aeruginosa	5	0.2	3	0.1	8	0.4
S. maltophilia	39	1.9	8	0.4	47	2.3
Carbapenems#	30	1.5	1	0.0	31	1.5
MRSA Drugs#	4	0.2	1	0.0	5	0.2
PCs#	111	5.5	11	0.5	122	6.1
Cephems#	109	5.4	4	0.2	113	5.6
Subgroup		%		%		%
Patients	1,525	87.5	217	12.5	1,742	100.0
	Median	(Quartile)	Median	(Quartile)	Median	(Quartile)
SOFA	0.3	(0-1)	0.2	(0-1)	0.4	(0-1)
Disease Severity$	3.2	(2-4)	3.1	(2-4)	3.3	(3-4)
CCI	4.9	(0-7)	4.7	(0-6)	6.4	(1-8)
MV day	8.1	(2-8)	7.8	(2-7)	10.3	(2-13)
EmOp (+):(-)	(+):105	(-):1,637	(+):101	(-):1,424	(+):10.3	(-):2

In a total of 113 patients lastly used cephem antimicrobials with VAE (Table 3), cefazolin (N=75, 66.4%) was the most frequent, followed by cefmetazole (N=33, 29.2%). They were mostly used for perioperative prophylaxis.

As for patient demographics, the median age of the 2,003 patients was 78 years, with an interquartile range of 69 to 84 years (Table 3). We collected key factors separating survival and mortality during MV, along with additional factors specific to each group (Table 3).

In terms of ventilation-days, the numbers and rates (per 1,000 ventilator days) for VAC, IVAC, and PVAP were 84 (4.7), 22 (1.2), and 6 (0.3), respectively (Table 2).

The timing of the last administration of antimicrobials relative to the end of MV, whether through extubation or death, is outlined as follows: 1) within 24 hours before the end of MV, 1,224 patients (61.1%) out of a total of 2,003 received their last antimicrobials; 2) on the day before the end of MV, 114 patients (7.5%) received their last antimicrobials out of a total of 1,523 patients who underwent MV for more than two days; 3) two days before the end of MV, no patients (0.0%) received their last antimicrobials out of a total of 1,158 patients who were on MV for more than three days.

Primary objective 1: mortality in final group

In a total of 2,003 patients of the final group, a univariate analysis demonstrated risk factors such as VAC for the outcome of mortality during MV (Table 4).

**Table 4:** Univariate logistic regression analysis showing odds ratios with 95% confidence intervals (CI). Cephems are associated with survival as are cold seasons (October–March, P = 0.007). Note: *, statistical significance (P < 0.05). MRSA, methicillin-resistant *Staphylococcus aureus*; ESBL, extended-spectrum beta-lactamase-producing microbes; *P. aeruginosa, Pseudomonas aeruginosa, *which may contain instability due to small numbers*; S. maltophilia, Stenotrophomonas maltophilia*; PCs, penicillins; VAC, ventilator-associated condition; IVAC, infection-related ventilator-associated complication; PVAP, probable ventilator-associated pneumonia.

Factor	Odds Ratio	95% CI		P
		Lower	Upper	
Age ≥ 75 Years	1.362	1.027	1.806	0.032*
Sex: Male with Female as Reference	1.038	0.79	1.364	0.791
Intensive Care	0.613	0.45	0.836	0.002*
Tracheostomy	0.762	0.493	1.18	0.223
Prone Position	2.334	1.088	5.006	0.030*
Pandemic	1.108	0.848	1.448	0.453
COVID-19	1.599	0.735	3.482	0.237
Influenza	0.506	0.066	3.863	0.511
Cold Season	0.781	0.598	1.02	0.069
MRSA	0.765	0.27	2.164	0.613
ESBL	1.424	0.166	12.236	0.104
P. aeruginosa	4.306	1.023	18.23	0.047*
S. maltophilia	1.474	0.681	3.191	0.325
Carbapenems#	0.234	0.032	1.723	0.154
MRSA Drugs#	1.78	0.198	15.995	0.607
PCs#	0.691	0.366	1.303	0.253
Cephems#	0.249	0.091	0.681	0.007*
VAC	2.168	1.277	3.681	0.004*
IVAC	2.114	0.773	5.781	0.145
PVAP	7.184	1.442	35.795	0.016*

**Figure 2:**
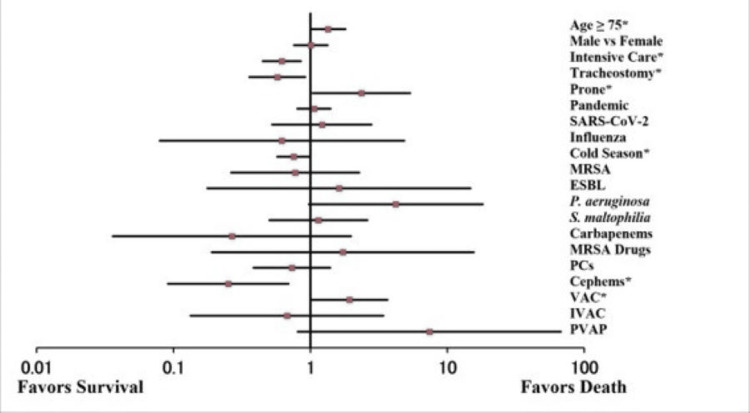
Final group (N=2,003) forest plot of multivariate logistic regression analysis showing odds ratios and 95% confidence intervals (dots and bars, respectively) for mortality during mechanical ventilation. Cold seasons (October–March) favor survival, as do cephems. Note: *, statistical significance (P < 0.05); MRSA, methicillin-resistant *Staphylococcus aureus*; ESBL, extended-spectrum beta-lactamase-producing microbes; *P. aeruginosa, Pseudomonas aeruginosa; S. maltophilia, Stenotrophomonas maltophilia*; PCs, penicillins; VAC, ventilator-associated condition; IVAC, infection-related ventilator-associated complication; PVAP, probable ventilator-associated pneumonia.

The multivariate analysis for the final group (Figure 2) identified several significant risk factors for mortality during MV. These included age ≥ 75 (odds ratio [OR] 1.343, 95% confidence interval [95% CI] 1.006-1.792, P = 0.045), prone positioning (OR 2.346, 95% CI 1.033-5.331, P = 0.042), and VAC (OR 1.926, 95% CI 1.017-3.645, P = 0.044). Regarding COVID-19, the multivariate analysis revealed that the pandemic had an OR of 1.059 (95% CI 0.801-1.400, P = 0.689), and SARS-CoV-2 positivity had an OR of 1.211 (95% CI 0.525-2.793, P = 0.654). However, neither of these findings was statistically significant (Figure 2).

We identified several protective factors that are associated with lower mortality during MV. These factors included intensive care (OR 0.617, 95% CI 0.448-0.850, P = 0.003), tracheostomy (OR 0.572, 95% CI 0.358-0.914, P = 0.02), cold seasons (OR 0.752, 95% CI 0.572-0.987, P = 0.040), and cephems (OR 0.25, 95% CI 0.091-0.688, P = 0.007) (Figure 2). 

Primary objective 2: mortality in subgroup

**Figure 3:**
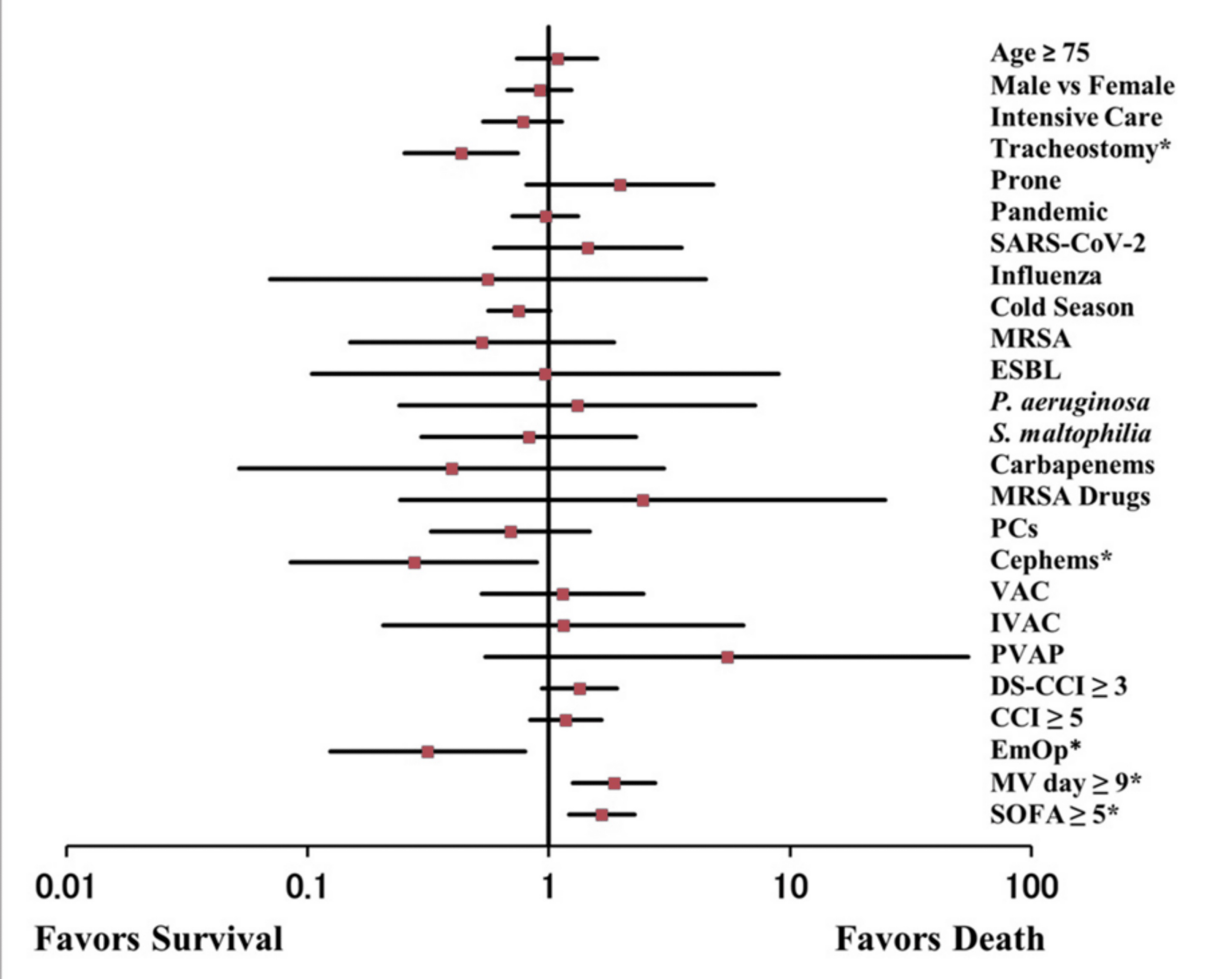
Subgroup (N=1,742) forest plot of multivariate logistic regression analysis showing odds ratios and 95% confidence intervals (dots and bars, respectively) for mortality during mechanical ventilation (MV). The subgroup includes data on sepsis-related organ failure assessment (SOFA) scores. Factors of MV days and SOFA are significant for mortality during MV. Cold season shows a marginal significance (P = 0.057) for survival. Note: *, statistical significance (P < 0.05); MRSA, methicillin-resistant *Staphylococcus aureus*; ESBL, extended-spectrum beta-lactamase-producing microbes; *P. aeruginosa, Pseudomonas aeruginosa; S. maltophilia, Stenotrophomonas maltophilia*; PCs, penicillins; VAC, ventilator-associated condition; IVAC, infection-related ventilator-associated complication; PVAP, probable ventilator-associated pneumonia; CCI, Charlson Comorbidity Index; DS-CCI, disease severity using CCI; EmOp, emergency operations.

In a study of 1,742 patients, after excluding those with missing values, we performed a multivariate logistic regression analysis to assess mortality during MV. The analysis revealed that an MV duration of nine or more days and a SOFA score of ≥5 were significantly associated with increased mortality during MV. Additionally, factors such as tracheostomy, the use of cephems, and emergency operations were linked to survival (Figure 3). Although the results for cold seasons were marginally significant (P = 0.057), they showed trends similar to those observed in the final group multivariate analysis (Figure 2).

Secondary objective 1: pandemic on mortality

The final group analysis indicated that the pandemic had no significant impact on mortality during MV. When we divided the pandemic period into three phases and compared it with the pre-pandemic era, we found that mortality during MV did not differ significantly among the four periods (Table 5).

**Table 5:** Mortality during mechanical ventilation (MV) in the pre-pandemic era, the early pandemic era, the vaccination era, and the endemic era. Chi-squared test of the mortality during MV for four eras shows no significance (P = 0.40).

Outcome	Pre-pandemic	Early	Vaccination	Endemic	Total
Survival	841	164	140	611	1,756
Death	112	20	27	88	247
%Mortality	-11.8	-10.9	-16.2	(12.6)	-12.3
Total	953	184	167	699	2,003

Secondary objective 2: seasonality on mortality

In the final group analysis of mortality during MV, we found that cold seasons were associated with an OR of 0.752 (P = 0.040), indicating a protective effect on mortality during MV. Similarly, cephems demonstrated a significant protective effect with an OR of 0.25 (P = 0.007).

To further explore the role of cold seasons, we conducted a correlation analysis involving six relevant factors. The results showed that cold seasons were marginally correlated with carbapenems and significantly correlated with cephems and influenza (P < 0.05, Table 6).

**Table 6:** Correlation analysis between seasonal factors and other pertinent factors in patients undergoing mechanical ventilation (N=2,003). Cold seasons (October–March) show a marginal correlation with carbapenems and a significant correlation with cephem antimicrobials and influenza. CC, correlation coefficients; MRSA, methicillin-resistant *Staphylococcus aureus*; PCs, penicillins; ESBL, extended-spectrum beta-lactamase-producing microbes; #, antimicrobials given lastly; *, significant.

Category		Antimicrobials				Microbes			
Factors		Carbapenems#	MRSA Drugs#	PCs#	Cephems#	MRSA	ESBL	Influenza	SARS-CoV-2
Cold Seasons	Pearson’s correlation coefficient	0.041	-0.032	-0.004	-0.066	0.027	0.02	0.061	-0.0001
	P	0.07	0.16	0.86	<0.01*	0.22	0.46	<0.01*	0.99

The correlation coefficients indicated that an increase in the use of carbapenems and a decrease in the use of cephems were associated with lower mortality during MV in the cold seasons. In terms of microbial correlation, neither MRSA nor ESBL-producing bacteria showed significant associations (Table 4).

**Figure 4:**
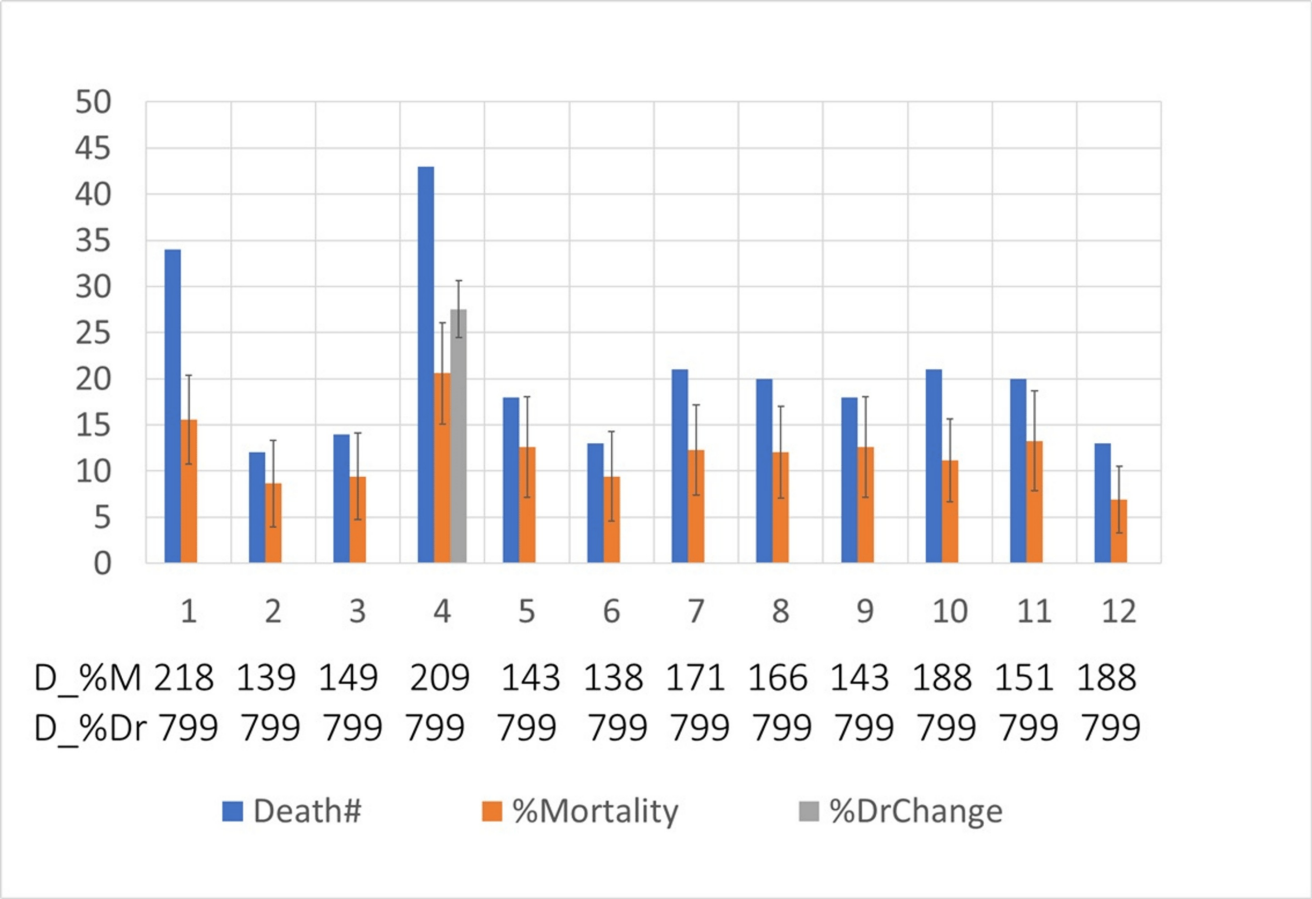
Monthly (on the x-axis) number of patients who died, mortality during mechanical ventilation (MV) per 100 patients (on the y-axis), and the rate of physicians' change (%DrChange) (on the y-axis) from 2013 to 2025 (N=2,003). In April (month 4), the number and the mortality during MV are the highest among 12 months (on the x-axis), corresponding to residents' and physicians’ transitions in Japanese hospitals. Note: D_, denominator; %M, mortality during MV; %Dr, rate of physicians' transit; tick mark, 95% confidence intervals.

In April, the highest number of patient deaths and mortality during MV occurred between 2013 and 2025 (Figure 4). A transition among physicians was noted in April, with a median rate of transition among all house staff physicians being 27.1% (interquartile range: 25.2% - 29.8%) (Figure 4).

## Discussion

Methodology

As early as 2017, Kerlin et al. [7] reported that diagnoses of ventilator-associated pneumonia were often unreliable and supported the diagnostic reliability of VAE, although VAE was found to be a poor predictor of pulmonary deterioration. Despite the reliability of VAE, a similar calculator developed at a single institution showed a sensitivity of 93.5% and a specificity of 100% when compared to a human abstractor [8].

Our in-house calculator is designed to perform calculations on the same intranet as the EHR. This system enabled a long-term analysis of VAE before and after the COVID-19 pandemic as follows.

COVID-19

Numerous studies have examined how the pandemic has influenced ventilator-associated pneumonia and, to some extent, VAE [9,10]. AlAhdal and colleagues [11] found no evidence that the pandemic led to an increase in device-associated infections like ventilator-associated pneumonia, attributing this to strict adherence to infection prevention and control measures.

In our series, neither the pandemic period nor positivity for SARS-CoV-2 was a significant risk factor for mortality during MV. Similarly, influenza did not significantly contribute to mortality during MV (Figure 1), as reported by Bechmann et al. [12]. The use of antiviral agents, such as remdesivir [13], which were prescribed early in the pandemic, may have helped reduce mortality during MV.

Microbiological and antimicrobial aspects

In both the final group and the subgroup analyzing mortality during MV, the multivariate analysis indicated a significant association between the use of cephems and improved survival (Figures 2, 3). From a microbiological perspective, neither viral infections (such as SARS-CoV-2 and influenza) nor bacterial infections showed a significant impact. The observed survival benefit associated with cephems can be attributed to their use in perioperative prophylaxis. Additionally, none of the drug-resistant microbes were associated with increased mortality during MV, in either the final group or the subgroup. This lack of significance is likely due to appropriate antimicrobial stewardship following the early detection of drug resistance.

The analysis of the correlation between the cold season and various factors indicated that the six cold months are significantly associated with influenza (P < 0.01) and with the use of carbapenems (P = 0.07). Carbapenems are primarily utilized for bacterial infections. However, their use as a preventive measure against drug-resistant bacteria during the cold season may have led to an increase in carbapenem prescriptions and, consequently, a reduction in mortality during MV. Despite this observation, the routine prophylactic use of carbapenems is not advisable from an antimicrobial stewardship perspective.

Age

Individuals aged ≥ 75, along with VAC positivity, were identified as significant risk factors, consistent with previous literature [14]. Shah and colleagues [15] reported that males and patients with co-morbidities, such as neurological disorders, are at an increased risk of developing ventilator-associated pneumonia, based on an analysis of 5,155,068 hospitalizations. Our study's multivariate analysis revealed that co-morbidities are a significant risk factor for mortality during MV, emphasizing the necessity of intensive care rather than general ward admission for these patients.

Seasons

Paradoxically, mortality during MV was found to be lower during the cold seasons (Table 2). This reduction in mortality during MV was associated with increased use of carbapenems and decreased use of cephems. However, the multivariate analysis indicated that the use of cephems was independently associated with lower mortality during MV. The use of broad-spectrum antimicrobials may have contributed to improved survival rates during MV. Rehman and colleagues [16] reported that broad-spectrum antimicrobials can help reduce mortality during MV. Antimicrobial resistance continues to be an important factor influencing mortality related to MV [17], presenting considerable challenges. Agrawal et al. [18] highlighted the importance of antimicrobial stewardship and resistance surveillance, which supports the findings of the current study that utilizes automated VAE surveillance.

Regarding academic seasons, Anioke and colleagues [19] conducted a study to examine the frequent occurrence of surgical site infections (SSIs) during warmer months, particularly around resident changeovers. They found that most SSIs in the United States occurred between July and August. Interestingly, this “July effect” was not observed in patients with acute respiratory distress syndrome receiving MV [20]. Similarly, Francis-Morel et al. [21] reported no significant impact on mortality rates among patients with diabetic ketoacidosis during July and August. In our study conducted in Japan, where the transition of residents and physicians occurs in April, we noted a possible “spring effect” that may have contributed to an increase in VAC (Figure 3).

Tracheostomy

One significant factor that reduces risk is the status of tracheostomy (Figure 1). Ochoa and colleagues [22] noted that tracheostomy could be a risk factor for mortality during MV. In our experience, tracheostomy facilitated easier sputum suction, reduced dead air space, and minimized colonization within airway devices. These aspects of MV mortality during MV warrant further investigation. Alkoheji and others [23] asserted that personalized decision-making supports the performance of tracheostomy, as the timing of the procedure does not affect the incidence of pneumonia or other complications.

Prone positioning

During the COVID-19 pandemic, prone positioning was introduced as a measure to improve oxygenation, and its effectiveness was confirmed in a randomized controlled trial [24]. However, there was a paradoxical increase in mortality associated with this practice (Figure 1). This unexpected outcome may have been influenced by selection bias, as prone positioning was primarily used on critically ill patients.

Limitations

This study had several limitations, including: 1) methodological shortcomings in evaluating the accuracy of the in-house calculator; 2) the inherent constraints of retrospective and single-center studies; and 3) unmeasured confounding and temporal changes over the 13-year period. For example, during the onset of COVID-19, prone positioning was encouraged to improve outcomes for patients with viral pneumonia. However, in our study, selection bias was more likely to affect those with severe conditions. Additionally, the supplementary dataset was historically biased, with missing values from the earlier years, leading to analysis primarily within a subgroup.

To generalize the current findings, such as the hypothetical seasonality observed, we await results from a multi-center study examining physicians' transit patterns and monthly mortality during MV in various institutions across Japan. Additionally, developing an in-house AI system for calculating VAEs may help to standardize our automated VAE calculator. However, the costs and server resource requirements pose challenges for this development.

## Conclusions

As to the primary objectives, we identified VAC, the first of three tiers of VAE, as a risk factor for mortality during MV.

Regarding the secondary objectives, we found that the COVID-19 pandemic and SARS-CoV-2 positivity were not associated with mortality during MV. Meanwhile, mortality during MV was associated with seasonal factors; however, whether antimicrobial prescribing patterns or physician turnover contributed requires further study.
